# Fucoidan Suppresses Hypoxia-Induced Lymphangiogenesis and Lymphatic Metastasis in Mouse Hepatocarcinoma

**DOI:** 10.3390/md13063514

**Published:** 2015-06-03

**Authors:** Hongming Teng, Yazong Yang, Hengyun Wei, Zundong Liu, Zhichao Liu, Yanhong Ma, Zixiang Gao, Lin Hou, Xiangyang Zou

**Affiliations:** 1 Department of Biotechnology, Dalian Medical University, Dalian 116044, China; E-Mails: thm0529@126.com (H.T.); yangyazong123.@126.com (Y.Y.); weihengyun@126.com (H.W.); harryblood1989@126.com (Z.L.); caredliu@126.com (Z.L.); m981765863@icloud.com (Y.M.); 18641548759@163.com (Z.G.); 2 College of Life Sciences, Liaoning Normal University, Dalian 116081, China

**Keywords:** fucoidan, lymphangiogenesis, hypoxia, HIF-1α, metastasis

## Abstract

Metastasis, the greatest clinical challenge associated with cancer, is closely connected to multiple biological processes, including invasion and adhesion. The hypoxic environment in tumors is an important factor that causes tumor metastasis by activating HIF-1α. Fucoidan, extracted from brown algae, is a sulfated polysaccharide and, as a novel marine biological material, has been used to treat various disorders in China, Korea, Japan and other countries. In the present study, we demonstrated that fucoidan derived from *Undaria pinnatifida* sporophylls significantly inhibits the hypoxia-induced expression, nuclear translocation and activity of HIF-1α, the synthesis and secretion of VEGF-C and HGF, cell invasion and lymphatic metastasis in a mouse hepatocarcinoma Hca-F cell line. Fucoidan also suppressed lymphangiogenesis *in vitro* and *in vivo*. In addition, accompanied by a reduction in the HIF-1α nuclear translocation and activity, fucoidan significantly reduced the levels of p-PI3K, p-Akt, p-mTOR, p-ERK, NF-κB, MMP-2 and MMP-9, but increased TIMP-1 levels. These results indicate strongly that the anti-metastasis and anti-lymphangiogenesis activities of fucoidan are mediated by suppressing HIF-1α/VEGF-C, which attenuates the PI3K/Akt/mTOR signaling pathways.

## 1. Introduction

Tumor metastasis is the dissemination of cancer cells from the initial site of primary tumor growth to distant organs, where they survive, proliferate and form secondary tumors. Metastasis is a complex process involving tumor cell invasion, adhesion, angiogenesis and lymphangiogenesis. Less attention has been focused on research into tumor lymphangiogenesis as opposed to angiogenesis.

Oxygen concentration changes and altered levels of oxygen-dependent or oxygen-independent growth factor lead to upregulation of hypoxia-inducible factor-1 alpha (HIF-1α) expression. Increasing evidence supports the hypothesis that the PI3K/Akt/mTOR pathway acts as a master switch controlling HIF-1α synthesis, thereby determining whether cells, especially tumor cells, grow and proliferate [1]. Therefore, PI3K/Akt/mTOR represents an attractive target for therapeutic intervention [2]. Matrix metalloproteinase-2, -9 (MMP-2, -9) and its endogenous inhibitor, tissue inhibitor of metalloproteinase-1 (TIMP-1), are involved in local tissue invasion, which is aided by the degradation of extracellular matrix (ECM) [3,4].

HIF-1α is a key transcription factor that activates the transcription of over 40 genes, including erythropoietin, glucose transporters, glycolytic enzymes, vascular endothelial growth factors (VEGFs) and other genes whose protein products increase oxygen delivery or facilitate metabolic adaptation to hypoxia, as well as promoting tumor invasion and metastasis and resistance to therapy [5,6,7,8]. Under hypoxic conditions, HIF-1α exists stably in the cytoplasm and is transferred to the nucleus, where it forms a heterodimer with the HIF-1β subunit to adapt the hypoxic environment [9,10]. In hepatocellular carcinoma (HCC), HIF-1α activation following extended hypoxia strongly correlates with an aggressive phenotype, metastasis and poor prognosis [11]. HIF-1α-induced expression of VEGF-C, a key downstream target of HIF-1α, promotes lymphangiogenesis and stimulates the proliferation and migration of lymphatic endothelial cells (LECs) [12]. Recent research has shown that the HGF, whose main function is the regulation of liver cell proliferation and the stimulation of the proliferation and migration of certain other epithelial cells, may facilitate lymphangiogenesis in a direct or an indirect manner, directly by activating the HGF receptor, also known as C-Met, and indirectly by activating the VEGF-C/VEGFR-3 signaling pathways [13]. NF-κB also has important tumor-promoting functions within inflammatory cells. Recent studies demonstrated that loss of IKKβ in myeloid cells prevents the development of lymph node metastases in colon cancer [14]. All of these pathways play critical roles in tumor progression by modulating HIF-1α synthesis, cell migration and invasion [15].

Fucoidan is vegetal fucose-containing polysaccharides extracted from brown algae and has many biological activities and few side effects. Fucose, uronic acid and sulfate have been identified in extracted and purified fucoidans [16,17,18,19]. Its biological activities include antioxidation, antitumor, antivirus, immunomodulation and as healing-impaired wound dressings [20,21,22,23,24,25]. However, there is no information about the fucoidan suppressing hypoxia-induced lymphangiogenesis and lymphatic metastasis, and its mechanism is ill defined.

In the present study, we evaluated the anti-tumor and anti-lymphangiogenesis effects of fucoidan on mouse hepatocarcinoma Hca-F cell line, which has high invasive and lymphatic metastasis potential, under hypoxic conditions. The study comprised three experiments: assessments of tumor growth, metastasis and lymphangiogenesis. The results will further reveal the mechanism of fucoidan anti-metastasis and anti-lymphangiogenesis under hypoxia.

## 2. Results and Discussion

### 2.1. Effect of Fucoidan on Cell Viability in CoCl_2_-Treated Hca-F Cells

**Figure 1:**
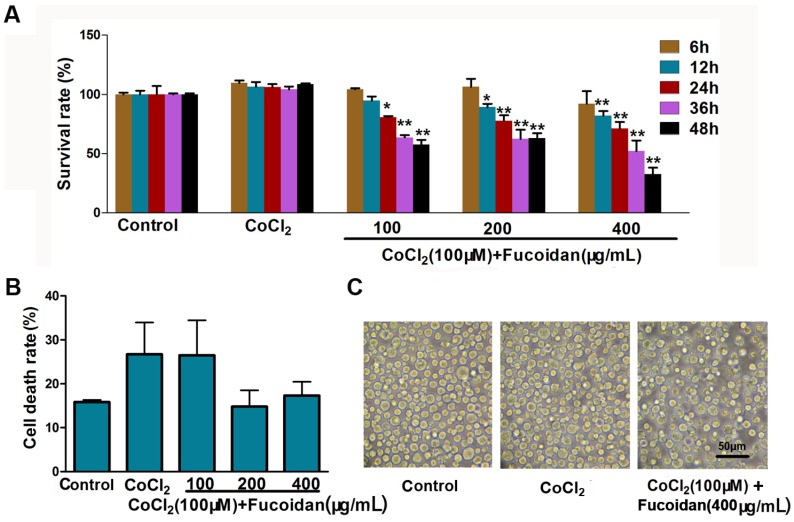
Effect of fucoidan on cell growth of CoCl_2_-treated Hca-F cells. Cells were treated with different concentrations of fucoidan (100, 200 and 400 µg/mL) for 6–48 h. Cells treated without fucoidan or CoCl_2_ served as the control group and normoxia group, respectively. (**A**) Cell viability was measured by the CCK-8 assay; (**B**) the cell death rate (*D*%) was counted and calculated; (**C**) the morphological change of fucoidan-treated cells was slightly dilated. Data represent the mean ± SD. * *p* < 0.05, ** *p* < 0.01, compared with the CoCl_2_ group.

Hca-F cells treated with 100 µM CoCl_2_, referred to as the hypoxia group in this study, showed a significant increase in cell viability, compared with the control group. The cells treated with fucoidan (100, 200 and 400 µg/mL) significantly decreased the viability compared with the CoCl_2_ group (Figure 1A). The cell death rate (*D*%) and the cell morphological change in different fucoidan concentration groups were not statistically significant compared to the control (Figure 1B,C).

### 2.2. Fucoidan Downregulates Expression and Activation of HIF-1α in CoCl_2_-Treated Hca-F Cells

**Figure 2:**
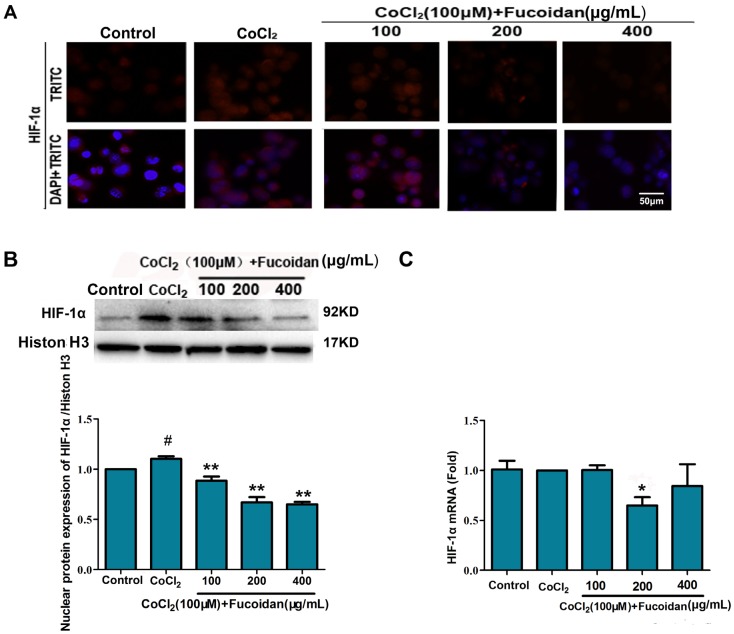
Fucoidan inhibits HIF-1α expression and stability. Nuclear translocation and expression of HIF-1α was detected by immunofluorescence assays (**A**); nuclear protein expression of HIF-1α (**B**) was measured by Western blotting; the mRNA expression level of HIF-1α (**C**) was tested by qRT-PCR, with GAPDH as the internal control. Data are presented as the mean ± SD of three independent experiments. ^#^ *p* < 0.05, compared with the control. * *p* < 0.05, ** p < 0.01, compared with the CoCl_2_ group.

To evaluate the effects of fucoidan on the expression and activation of HIF-1α under hypoxic conditions, Western blotting and immunofluorescence staining were performed. After exposure to CoCl_2_, HIF-1α mRNA and protein levels were significantly increased. However, for cells treated with fucoidan, total and nuclear protein levels of HIF-1α decreased. Immunofluorescence assays revealed that the activity and nuclear translocation of HIF-1α were significantly inhibited by fucoidan (Figure 2A–C). These results suggested that fucoidan can inhibit HIF-1α expression and nuclear translocation.

### 2.3. Fucoidan Inhibits Cell Invasion in CoCl_2_-Treated Hca-F Cells

**Figure 3:**
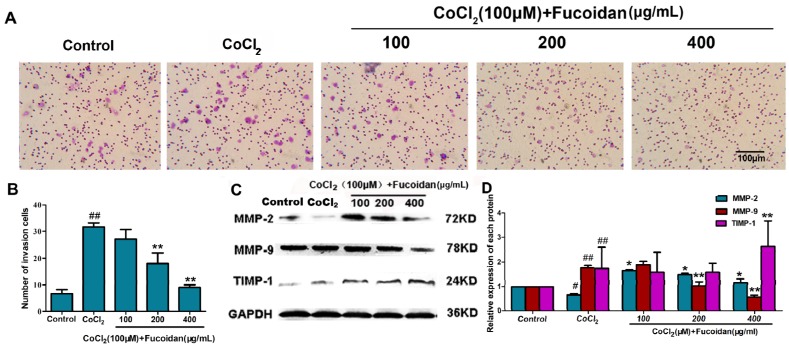
Fucoidan inhibits CoCl_2_-treated Hca-F cell invasion. The invaded Hca-F cells treated with fucoidan under hypoxic conditions were stained and counted (**A**,**B**); Western blot showing that fucoidan decreased MMP-2 and MMP-9 expression and increased TIMP-1 expression in a concentration-dependent manner under hypoxic conditions (**C**,**D**). The quantitative results were analyzed by Image Lab 4.0 software, and GAPDH was used as the internal control. The relative expression quantity of the each protein compared with the control, respectively. Data are the mean ± SD of three independent experiments. ^#^ *p* < 0.05, ^##^ *p* < 0.01, compared with the control. * *p* < 0.05, ** *p* < 0.01, compared with the CoCl_2_ group.

In the cell invasion assay, the invasive activity of Hca-F cells treated with fucoidan was suppressed under hypoxic conditions. The invasion rate of fucoidan-treated cells (400 μg/mL, 24 h, 20% ± 2%) was significantly lower compared with the hypoxia control (62.67% ± 2.52%) (Figure 3A,B). The relative expression quantity of invasion-related proteins, including MMP-2 and MMP-9, and their specific inhibitor, TIMP-1, were detected by Western blotting. The expression of MMP-9 was significantly downregulated, and that of TIMP-1 was upregulated in Hca-F cells treated with fucoidan under hypoxia conditions (Figure 3C,D).

### 2.4. Fucoidan Inhibits VEGF-C and HGF Production in CoCl_2_-Treated Hca-F Cells

**Figure 4:**
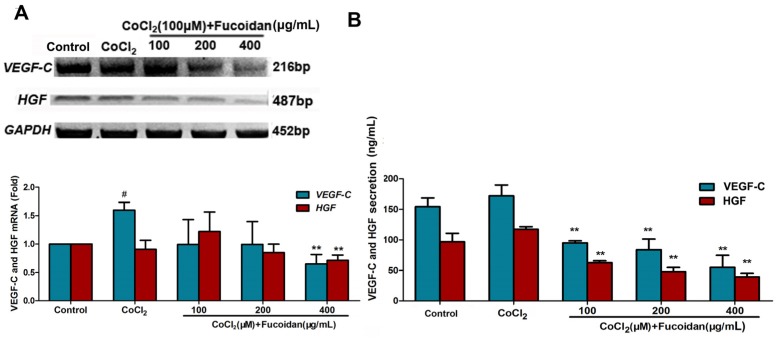
Fucoidan decreased the production and expression of VEGF-C and HGF in the cells under hypoxic conditions. The HGF and VEGF-C mRNA levels were detected using a qRT-PCR assay (**A**); VEGF-C and HGF protein concentrations in the conditioned medium were determined using a quantitative ELISA kit (**B**). Each value represents the mean ± SD. ^#^ *p* < 0.05, compared with the control. ** *p* < 0.01, compared with the CoCl_2_ group.

After observing the anti-metastatic effect of fucoidan in Hca-F cells, the underlying molecular mechanism for this inhibition was evaluated using ELISA assays. The results showed that the levels of VEGF-C and HGF proteins in the culture medium of the cells treated with fucoidan in hypoxia were lower compared with those of the control. Furthermore, mRNA levels of VEGF-C and HGF were downregulated in the presence of fucoidan (400 µg/mL) (Figure 4).

### 2.5. Fucoidan Downregulates the Expression of VEGFR-3 and C-Met in CoCl_2_-Treated Hca-F Cells

**Figure 5:**
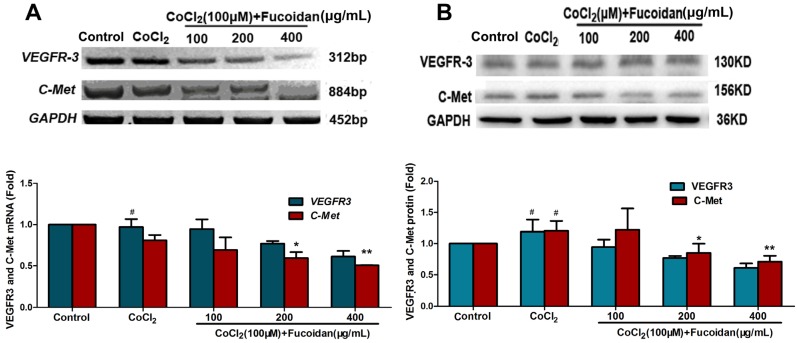
Effect of fucoidan on protein expression of VEGFR-3 and C-Met in CoCl_2_-treated cells. VEGFR-3 and C-Met expressions significantly decreased following fucoidan treatment in CoCl_2_-treated cells, as assessed by qRT-PCR (**A**) and Western blotting (**B**). Each value represents the mean ± SD, obtained from three independent experiments. ^#^ *p* < 0.05, compared with the control. * *p* < 0.05, ** *p* < 0.01, compared with the CoCl_2_ group.

To determine whether fucoidan inhibits receptor tyrosine kinase expression in CoCl_2_-treated cells, VEGFR-3 and C-Met mRNA and protein levels were detected using Western blotting and qRT-PCR assays. Upon treatment with fucoidan, both the mRNA and protein levels of VEGFR-3 and C-Met decreased in Hca-F cells under hypoxic conditions (Figure 5).

### 2.6. Fucoidan Modulates Hypoxia-Related Multiple Signaling Pathways

**Figure 6:**
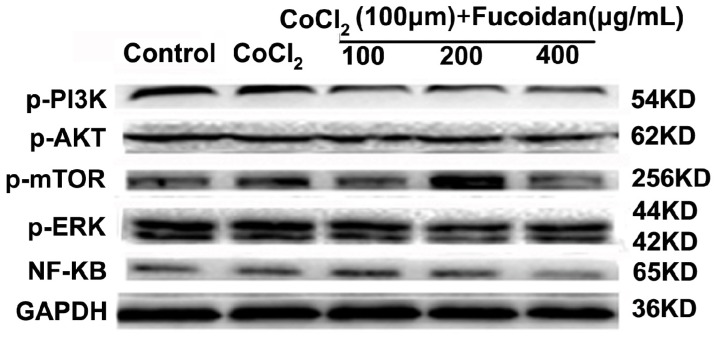

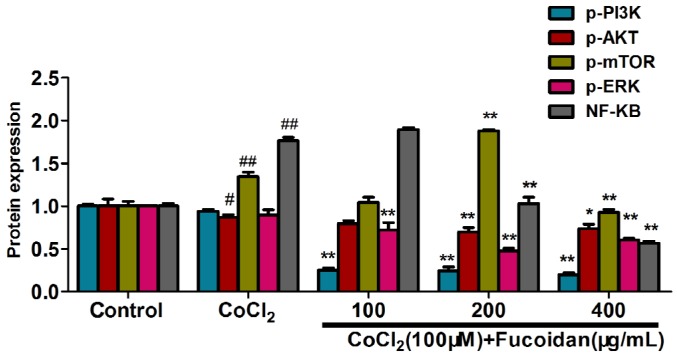
Effect of fucoidan on the activation of hypoxia-related signaling pathways. The levels of p-PI3K, p-AKT, p-mTOR, p-ERK and NF-κB were decreased, which indicated that the activation of PI3K/AKT/mTOR, ERK and NF-κB in CoCl_2_-treated cells was downregulated. Each value represents the mean ± SD. ^#^ *p* < 0.05, ^##^ *p* < 0.01, compared with the control. * *p* < 0.05, ** *p* < 0.01, compared with the CoCl_2_ group.

To characterize the mechanism of the inhibitory effect of fucoidan on Hca-F cells under hypoxia conditions, the activities of the PI3K/Akt/mTOR, ERK and NF-κB signaling pathways were assessed. Western blot analysis showed that p-PI3K, p-AKT, p-mTOR, p-ERK and NF-κB protein levels were downregulated in Hca-F cells treated with fucoidan under hypoxia conditions (Figure 6).

### 2.7. Fucoidan Inhibits Metastasis of Hca-F Cells in Vivo

**Figure 7:**
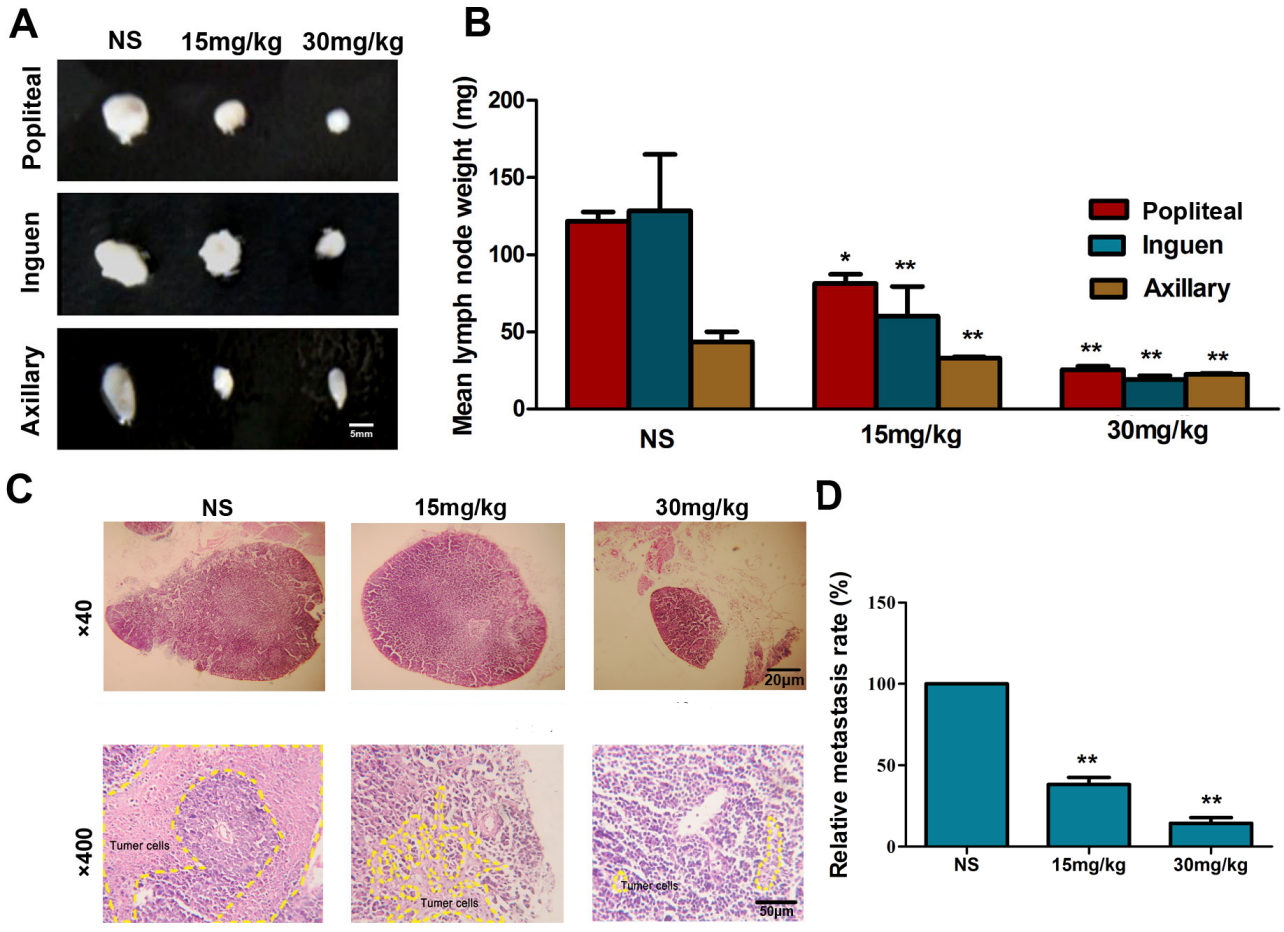
Fucoidan inhibits the cell metastasis *in vivo.* Popliteal, inguinal and axillary lymph nodes were removed and photographed (**A**); shown with their average weight, respectively (**B**); axillary lymph nodes with H.E staining (**C**); the relative metastasis rate (**D**). Note that the tissue structures of the metastatic lymph node were obscured in the control; however, the structure of the lymph node tissue was obvious in mice treated with fucoidan. Data are the mean ± SD. * *p* < 0.05, ** *p* < 0.01, compared with the normal saline (NS) group.

Hca-F engrafted mice treated with fucoidan were sacrificed, and lymph nodes were isolated and weighed. The popliteal, inguinal and axillary lymph nodes of every group are shown in Figure 7A,B. The mean lymph node weight in mice inoculated with Hca-F cells followed by fucoidan treatment was lower compared to the normal saline (NS) group (*p* < 0.05, *n* = 6). Histological examination of the lymph nodes showed that Hac-F cells infiltration could be attenuated by fucoidan (Figure 7C,D).

### 2.8. Fucoidan Inhibits the Expression of HIF-1α and VEGF-C and Lymphangiogenesis in Vivo

**Figure 8:**
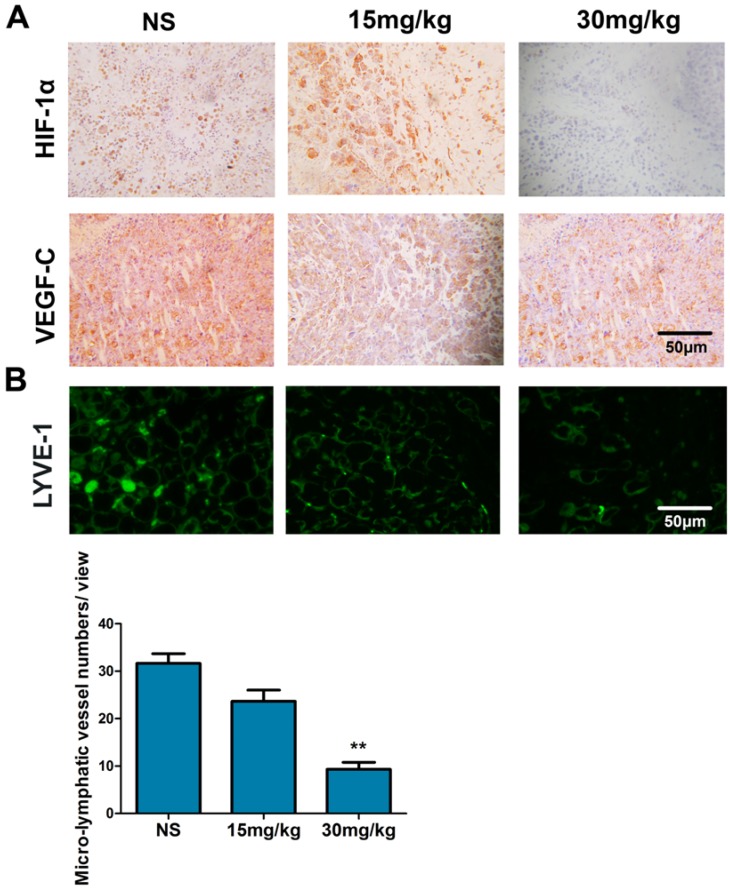
Fucoidan inhibits the expression of HIF-1α and VEGF-C and lymphangiogenesis *in vivo*. Immunohistochemical staining for HIF-1α and VEGF-C (**A**). Immunofluorescence staining of LYVE-1 was used to evaluate MLVD. Representative photomicrographs of LYVE-1^+^ peritumoral lymphatic vessels surrounding the tumor (**B**). ** *p* < 0.01, compared with the NS group.

In tumor tissues treated with fucoidan, the expressions of HIF-1α and VEGF-C were lower compared with the control (Figure 8A). LYVE-1^+^ cells were absent from Hac-F tumors, but peritumoral staining of irregularly-shaped thin-walled micro-lymphatic vessels for LYVE-1 was evident. Treatment with fucoidan (30 mg/kg) significantly decreased peritumoral MLVD in tumor tissues compared with the NS group (Figure 8B) (*p* < 0.01).

### 2.9. Fucoidan Inhibits Lymphangiogenesis in Vitro

**Figure 9:**
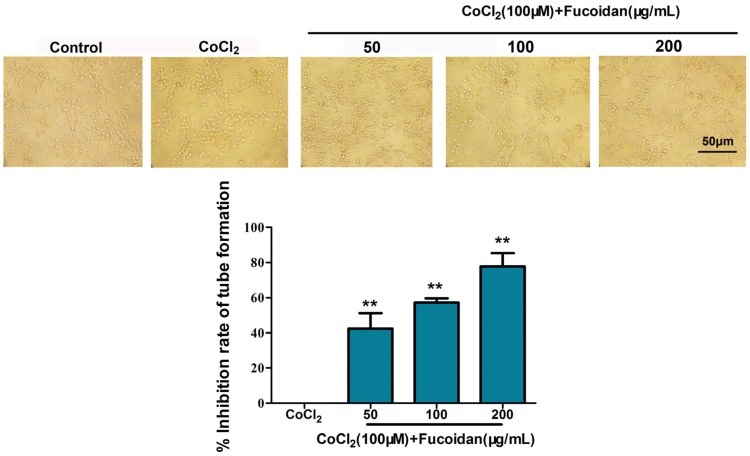
Fucoidan inhibits lymphangiogenesis *in vitro.* Tube formation in mouse lymphatic endothelial cells (mLEC) was suppressed by fucoidan (50, 100 and 200 µg/mL) for 48 h in a hypoxic environment. ** *p* < 0.01, compared with the CoCl_2_ group.

The effect of fucoidan on tube formation in mouse lymphatic endothelial cells (mLECs) was then observed *in vitro*. The tube formation ability of mLECs was inhibited by fucoidan under hypoxic conditions (Figure 9) (*p* < 0.01).

### 2.10. Discussion

Hepatocellular carcinoma is the third leading cause of death from tumors worldwide [26]. Although successful curative hepatectomy has significantly improved survival, the prognosis remains poor because of tumor invasiveness, frequent intrahepatic spread and extrahepatic metastasis.

Hypoxia (low oxygen tension) is a critical feature of the tumor microenvironment that promotes invasion and metastasis, as well as resistance to therapy. HIF-1α is a key regulating factor of the response to hypoxia. Under hypoxic conditions, HIF-1α is stabilized because of the lack of O_2_ and dimerizes with HIF-1β; it then interacts with the coactivator CBP/p300 to bind to the hypoxia response element (HRE, 5′-G/ACGTG-3′) on the promoter region of various target genes [27,28,29,30]. CoCl_2_, as a hypoxia-mimicking agent, has been widely used to enhance the expressions of a set of hypoxia-responsive genes [31]. In the present study, cell viability increased slightly, and HIF-1α mRNA and nuclear protein levels also increased after Hca-F cells were treated for 6–48 h with 100 µM of CoCl_2_.

Specifically, fucoidan from *U. pinnatifida* sporophylls has a higher sulfate level and unique bioactivities [32]. Our previous studies found that fucoidan prevents tumor growth, induces cell apoptosis and has anti-angiogenesis and anti-metastatic effects [21,22,25]. However, the mechanisms of fucoidan’s anti-metastatic and anti-lymphangiogenesis bioactivities have not been determined. We observed that treatment with fucoidan decreased HIF-1α expression and its nuclear translocation in CoCl_2_-induced cells in a fucoidan dose-dependent manner.

HIF-1α modulates downstream target genes, including *VEGF-C* and *HGF*, which act on tumor cells through autocrine and paracrine pathways, enhancing hepatocarcinoma growth, inducing lymphangiogenesis and promoting lymphatic metastasis in the hypoxic microenvironment [33,34]. Our results demonstrated that, in the hypoxic environment, fucoidan inhibited the expression and secretion of VEGF-C and HGF, which are the target genes of HIF-1α. Meanwhile, HIF-1α also upregulated the expressions of MMP-2 and MMP-9, which induce ECM breakdown and promote metastasis by lymph nodes [28]. In this study, fucoidan inhibited the cells’ invasion in a manner related to decreasing MMP-9 and increasing TIMP-1 expression, leading to suppression of ECM breakdown and migration in the hypoxic environment.

VEGF-C is a dimeric glycoprotein, and it is a ligand for its cognate receptor, VEGFR-3, playing a pivotal role in LECs’ proliferation and the lymphangiogenesis of cancer [31]. Hypoxic microenvironments contribute to lymphangiogenesis by secreting VEGF-C and other mediators. VEGF expression is induced by HIF-1α; therefore, whether fucoidan could suppress VEGF transcription and activation in the Hca-F cells was further examined. As expected, fucoidan decreased hypoxia and HIF-1α-induced VEGF-C; meanwhile, VEGFR-3, a specific receptor of VEGF-C, also decreased. HGF also promotes lymphangiogenesis and the growth of endothelial cells (ECs) by interacting with the VEGF-C pathway. Increased VEGF-C expression in response to hypoxia increased C-Met expression. C-Met activation induced metastasis in xenograft models of different tumors and in transgenic mice that overexpressed C-Met or HGF [35,36]. Our results showed that fucoidan could decrease C-Met and HGF expressions, suggesting that fucoidan regulates HIF-1α-induced HGF expression and metastasis via inhibiting HGF and C-Met, which is closely related to inhibited Hca-F cell growth and lymphangiogenesis.

The HIF-1α protein synthesis-related PI3K-AKT-mTOR signaling pathway is one of the three major signaling pathways that have been identified as important in cancer. mTOR is a key kinase downstream of PI3K/AKT, which regulates tumor cell proliferation, survival and angiogenesis. Recent data suggested that the PI3K-AKT-mTOR signaling pathway plays an important role in cancer stem cell self-renewal and resistance to chemotherapy or radiotherapy, which is believed to be the root of treatment failure and cancer recurrence, as well as metastasis [37]. P-AKT activates IκB kinase (IKKα), leading to inhibition of NF-κB degradation by IκB, allowing NF-κB to be transferred into the nucleus from the cytoplasm, where it activates its target genes and promotes cell survival. Activation of p-PI3K through stimulation of receptor tyrosine kinase (RTK) phosphorylation leads to the activation and inhibition of several targets, resulting in cellular growth, survival and proliferation through various mechanisms. Translation of the HIF-1α protein is regulated by intracellular signaling pathways, including the ERK and PI3K/Akt pathways. Our data showed that the phosphorylation of the PI3K/Akt/mTOR cascade was significantly inhibited by fucoidan in hypoxic cells, which could be explained if HIF-1α expression resulted in increased HIF-1α protein that suppressed the PI3K/Akt/mTOR signaling pathway in cells treated with CoCl_2_. Meanwhile, our findings indicated that fucoidan inactivated the ERK and NF-κB signaling pathways, which suggested that fucoidan regulates HIF-1α and VEGF expression via inhibiting NF-κB-independent PI3K/Akt/mTOR and ERK signaling pathways.

Lymphangiogenesis is a key factor not only for tumor growth, but also for tumor metastasis. Observations from clinicopathological studies have suggested that lymphatic vessels serve as the primary route for the metastatic spread of tumor cells to regional lymph nodes. The MLVD is associated with lymphatic nodal metastases. Recent studies in animal models have provided convincing evidence that tumor lymphangiogenesis facilitates lymphatic metastasis [38,39]. As expected, fucoidan exerts a potent anti-lymphangiogenic effect *in vitro* and *in vivo*, evidenced by the inhibition of tube formation of mouse LECs in Matrigel plus in the presence of hypoxia. Similarly, in a mouse Hca-F hepatocarcinoma metastasis model, administration of fucoidan significantly reduced the number of tumor cell metastasizing to lymph nodes and decreased the MLVD in tumors, as evidenced by decreased HIF-1α and VEGF-C expression.

## 3. Materials and Methods

### 3.1. Reagents and Antibodies

RPMI 1640 medium, penicillin-streptomycin and fetal bovine serum (FBS) were purchased from HyClone Cell Culture (Thermo Fisher Scientific, Carlsbad, CA, USA). Matrigel and cobalt chloride (CoCl_2_) were purchased from Sigma (St. Louis, MO, USA). The Cell Counting Assay Kit-8 solution (CCK-8) was purchased from Dojindo (Kumamoto, Japan). Rabbit anti-VEGF-C and anti-VEGFR-3 antibodies were purchased from Santa Cruz Biotechnology (Santa Cruz, CA, USA). Rabbit anti-phosphorylated (p)-PI3K, anti-p-Akt and anti-NF-κB antibodies were purchased from Bioworld Technology (Nanjing, China). Rabbit anti-MMP-2, anti-MMP-9 and anti-GAPDH antibodies were purchased from KeyGen Biotech (Nanjing, China). Rabbit anti-TIMP-1 antibody was purchased from Solarbio Science & Technology (Beijing, China). Mouse anti-GAPDH antibody was purchased from KangChen Bio-tech (Shanghai, China). Horseradish peroxidase-conjugated anti-rabbit and anti-mouse IgG were purchased from Thermo Fisher Scientific.

### 3.2. Purification and Analyses of Fucoidan

The crude fucoidan used in this study was kindly provided from Dalian Aquaculture Group Co., Ltd. (Dalian, China). The *U. pinnatifida* sporophyll was collected from the coast of Dalian, Liaoning province, China, in March 2012, which was harvested free from environmental and radioactive contamination. Crude fucoidan was extracted with distilled water at 80 °C for 1 h [23]. The fucoidan was purified as previously reported [20]. The fucoidan (purity > 90%) contained 68.37% carbohydrate, 21% sulfates and 10.89% uronic acid; the protein content was merely 0.13%. Its molecular weight was approximately 10.4356 × 10^4^ Da.

### 3.3. Cell Culture and Treatments

The mouse hepatocarcinoma cell line Hca-F, with high invasion and lymphatic metastasis potential, was established and stored by the Department of Pathology, Dalian Medical University [40]. The Hca-F cells were maintained in RPMI 1640 medium supplemented with 10% fetal bovine serum (FBS), 100 U/mL penicillin and 100 µg/mL streptomycin in a humidified incubator containing 5% CO_2_ at 37 °C. Cells were treated with 100 µM CoCl_2_ to mimic hypoxic conditions.

### 3.4. Cell Viability Assay

Cell viability was determined by a CCK-8 assay. In brief, Hca-F cells (1.0 × 10^5^ cells/well, in 100 µL medium) seeded in 96-well microtiter plates were exposed to fucoidan at given concentrations for 6–48 h and treated simultaneously with CoCl_2_ in final volumes of 200 µL. Ten microliters of CCK-8 were added to each well and incubated for 30 min, and the absorbance at 490 nm was measured using a Multiskan Ascent microplate photometer (Thermo Fisher Scientific, Carlsbad, CA, USA). Cell viability was expressed as the percentage of the absorbance of treated cells relative to the absorbance of control cells. Similarly, cell death was identified by trypan blue staining for 24 h. The numbers of total cells and dead cells were counted. The cell death rate (*D*%) was calculated using the following equation: *D*% = (*C*_death cell_/*C*_total cell_) × 100%.

### 3.5. Cell Invasion Assay in Vitro

For invasion assays, Hca-F cells treated with fucoidan (0, 100, 200 and 400 µg/mL) and CoCl_2_ (100 µM) for 24 h were suspended in serum-free medium, 1 × 10^5^ cells were plated to the top chambers of transwell coated with Matrigel. Then, the lower chambers were added with 500 mL medium containing 20% FBS as a chemoattractant. After incubation for 18 h, the cells that did not invade or migrate to the upper wells were removed with a cotton swab. Cells passed through the membrane on the lower surface were fixed with 70% ethanol, stained with crystal violet for 30 min, observed with a microscope and counted with five fields assessing cell invasion rate.

### 3.6. RT-PCR Analysis

Total RNA was isolated from Hca-F cells using the TRIzol reagent (Invitrogen, Carlsbad, CA, USA), according to the manufacturer’s instructions. The concentration and purity of RNA were determined at 260/280 nm using a Nanodrop spectrophotometer (Thermo Scientific, Rockford, IL, USA). Total RNA (1.0 µg) was then reverse-transcribed using the SuperScript III first-strand synthesis system RT-PCR kit (Invitrogen, Carlsbad, CA, USA), according to the manufacturer’s protocol. Additionally, the target cDNAs were amplified using primer pairs for *hif-1*α, *vegfr-3*, *vegf-c*, *hgf* and *c-met*. *Gapdh* was used as the internal standard. The sequences of the forward and reverse primers and the product lengths are shown in Table 1. All primers were synthesized by Takara (Dalian, China). The relative quantitation of mRNA expression levels was determined using the relative standard curve method according to the manufacturer’s instructions.

**Table 1:** Primers used in qRT-PCR.

Gene		Primers Sequence (5′-3′)	Product
*hif-1*α	Forward Reverse	AAACCTGGCAATGTCTCC TGCCTTAGCAGTGGTCGT	350 bp
*vegfr-3 *	Forward Reverse	CTTTCCAATCTCTTCGTCG GAACCGCTGAATCCCAT	312 bp
*vegf-c *	Forward Reverse	GTCCATCCACCATGCACTTG GCTCATCTACGCTGGACACA	216 bp
*hgf *	Forward Reverse	GGACCAGCAGACACCACA TTTCCCATTGCCACGATA	487 bp
*c-met *	Forward Reverse	GTGCCCGAAGTGTAAGTCCA TCTCGTCATGAGCTCCCAGA	884 bp
*gapdh *	Forward Reverse	ACCACAGTCCATGCCATCAG TCCACCACCCTGTTGCTGTA	452 bp

### 3.7. Western Blotting

Cells treated with or without fucoidan under normoxic or hypoxic conditions were collected, washed twice with chilled PBS and lysed in RIPA buffer (Sigma, St. Louis, MO, USA) or using a Nucleoprotein cytoplasm protein extraction kit (KeyGen Biotech, Nanjing, China), according to the manufacturer’s instructions. Equal amounts of protein extract were subjected to 12% SDS-PAGE gels and transferred to nitrocellulose (NC) membranes (Solarbio, Beijing, China). The primary antibodies, including dilution conditions, are as follows: rabbit anti-p-PI3K (1:500), p-Akt (1:500), p-mTOR (1:500), NF-κB (1:500) and anti-His or mouse anti-GAPDH as an internal control for HIF-1α or other proteins.

### 3.8. Immunofluorescence Staining

Cells were fixed with 4% paraformaldehyde, blocked with 10% BSA and incubated with antibody anti-HIF-1α (1:200) overnight at 4 °C in a humidified container. Immunolabeling was revealed by FITC-conjugated secondary antibodies. Nuclei were counterstained with DAPI. Immunofluorescence was observed under a fluorescence microscope (Leica, Solms, Germany).

### 3.9. Enzyme Linked Immunosorbent Assay

Hca-F cells were incubated with fucoidan (0, 100, 200 and 400 µg/mL) and CoCl_2_ (100 µM) for 24 h, and the collected supernatants were detected by ELISA, as previously reported [24]. Ninety six-well plates were coated with polyclonal anti-VEGF-C and anti-HGF (1:1000) antibodies, respectively. The plates were read on a Multiskan Ascent photometer (Thermo Fisher Scientific, Carlsbad, CA, USA) at 450 nm.

### 3.10. Tumor Metastasis Analysis in Vivo

All studies involving mice were approved by the Animal Care and Use Committee of Dalian Medical University. Eighteen eight-week-old male 615 mice (obtained from the Experimental Animal Center) were inoculated subcutaneously in the foot-pad with Hca-F cells (1.6 × 10^6^, 40 µL) and then were assigned equally to three groups (6 animals per group). After 48 h, 200 μL of normal saline (NS, as the control group) or fucoidan (15 mg/kg and 30 mg/kg, 200 µL) were injected into the abdominal cavity once every day. Mice were sacrificed 3 weeks later. Popliteal, inguinal and axillary lymph nodes and tumors *in situ* were isolated, weighed, sectioned and stained with hematoxylin and eosin (HE) and subjected to immunohistochemical and immunofluorescence assays, using anti-HIF-1α, anti-VEGF-C and anti-LYVE-1 (as a biomarker for lymphatic endothelial cells) antibodies, respectively.

### 3.11. Tube Formation Assay

The mouse LECs (mLECs) were induced by intraperitoneal injection of incomplete Freund’s adjuvant [41]. The formation of lymphatic tube-like structures by mLECs was assessed in a Matrigel-based assay, as previously described [42]. The mLECs were trypsinized, resuspended in DMEM medium containing fucoidan (0, 50, 100 and 200 μg/mL) with CoCl_2_ (100 µM) and then seeded onto the Matrigel at a density of 1 × 10^4^ cells/well for 48 h. Tube formation was examined and photographed by phase-contrast microscopy from five different fields (100×). Inhibition of tube formation was calculated by the equation: *I*% = [1 − (tube quantity_treat_/tube quantity_control_)] × 100%.

### 3.12. Statistical Analysis

Data were expressed as the mean ± standard deviation (SD). Data were subjected to statistical analysis by one-way analysis of variance (ANOVA). *p* < 0.05 was considered statistically significant.

## 4. Conclusions

Taken together, our findings indicate, for the first time, that fucoidan inhibits HIF-1α expression/nuclear translocation and its downstream signaling pathways; inhibits VEGF-C and HGF secretion and their receptors, VEGFR-3 and C-Met expressions; and decreases MMP-9 and increases TIMP-1 expression in Hca-F cells under hypoxia. Moreover, hypoxia-induced lymphangiogenesis and tumor lymphatic metastasis were markedly reduced by fucoidan treatment. These functions are mediated by suppressing HIF-1α protein accumulation/nuclear translocation and synthesis via the PI3K/AKT/mTOR signaling pathway, as well as by decreasing the expression of HIF-1α’s target genes, including VEGF-C, HGF and MMP-9, in hypoxia cells. Our results provide a possible mechanism to explain the anti-lymphangiogenic and antitumor effects of fucoidan and suggest that fucoidan could be considered as a potential pharmacological agent to prevent tumor metastasis.

## Abbreviations

HIF-1α, hypoxia-inducible factor-1 alpha; PI3K, phosphatidylinositol 3 kinase; AKT, cellular homolog of v-akt oncogene, a serine/threonine protein kinase; mTOR, mammalian target of rapamycin; VEGF-C, vascular endothelial growth factor-C; VEGFR-3, vascular endothelial growth factor receptor-C; HGF, hepatocyte growth factor; NF-κB, nuclear factor-κB; ERK, extracellular signal-regulated kinase; LYVE-1, lymphatic vessel endothelial hyaluronan receptor-1; MLVD, microlymphatic vessel density.
